# Analysis of Different Early Strength Agents on the Performance of Prefabricated UHPC

**DOI:** 10.3390/ma17112481

**Published:** 2024-05-21

**Authors:** Xiaohu Wu, Lien Hu, Fucheng Guo, Xiaomin Li

**Affiliations:** 1 School of Civil Engineering, Lanzhou Jiaotong University, Lanzhou 730070, China; ldxyhle@gmail.com (X.W.); fcguo@chd.edu.cn (F.G.); 2 Gansu Provincial Department of Transportation, Lanzhou 730030, China; 3 Gansu Provincial Transportation Planning Survey and Design Institute Co., Ltd., Lanzhou 730030, China; 4 Department of Civil Engineering, Kyungsung University, Busan 48445, Republic of Korea; 5 Key Laboratory for Special Area Highway Engineering of Ministry of Education, Chang’an University, Xi’an 710064, China; 6 Gansu-Highway Traffic Construction Group Co., Ltd., Lanzhou 730050, China

**Keywords:** ultra-high-performance concrete (UHPC), early strength agents, Ca(HCO_2_)_2_, Al_2_(SO_4_)_3_, early strength, microstructure

## Abstract

Precast ultra-high-performance concrete (UHPC) has emerged as indispensable in the engineering sector due to its cost-effectiveness and superior performance. Currently, precast UHPC grapples with challenges pertaining to slow setting times and insufficient early strength, largely attributed to its high water-reducing agent content. Effective utilization of early strength agents to augment UHPC’s early strength is pivotal in addressing this issue. This study investigates the efficacy of two distinct concrete early strength agents, namely calcium formate (Ca(HCO_2_)_2_) and aluminum sulfate (Al_2_(SO_4_)_3_). A UHPC system with a water/cement ratio of 0.17 was used; both single and compound doping experiments were conducted using varied dosages of the aforementioned early strength agents. Our results show that both early strength agents significantly reduce setting time and enhance early strength at appropriate dosages. Specifically, the addition of 0.3% Ca(HCO_2_)_2_ led to a 33.07% decrease in setting time for UHPC. Moreover, the incorporation of 0.3% Ca(HCO_2_)_2_ and 0.5% Al_2_(SO_4_)_3_ resulted in a strength of 81.9 MPa at 1.5 days, representing a remarkable increase of 118.4%. It is noteworthy that excessive use of Ca(HCO_2_)_2_ inhibits the hydration process, whereas an abundance of Al_2_(SO_4_)_3_ diminishes the early strength effect. Simultaneously, this article provides recommendations regarding the dosage of two distinct early strength agents, offering a novel solution for expediting the production of prefabricated UHPC with a low water/cement ratio and high water-reducing agent content.

## 1. Introduction

UHPC stands out as a seminal advancement in concrete material research. UHPC exhibits superior compressive and tensile strength, as well as enhanced crack resistance and durability, compared to conventional concrete [1]. These remarkable attributes have facilitated the widespread utilization of UHPC in critical projects and components, yielding outstanding outcomes [2,3]. Despite the clear advantages of UHPC over traditional concrete, it exhibits certain shortcomings. UHPC exhibits slower early strength development, consequently prolonging the time required to achieve the design strength and extending the engineering cycle. The unique characteristics of UHPC necessitate stringent construction technology requirements, demanding specialized training and qualifications for efficient execution, potentially amplifying project complexity and cost. Furthermore, the high strength and low water/cement ratio of UHPC lead to its relatively poor plasticity, potentially requiring additional handling and processing in select construction applications, thus elevating engineering complexity [4]. Cast-in-place UHPC is vulnerable to environmental factors, leading to quality discrepancies [5,6]. For critical components, prefabricated assembly stands out as the optimal choice [7,8,9]. Precast UHPC components typically require customization and fabrication to meet precise design criteria and engineering standards, potentially increasing both design and production expenditures. Nevertheless, precast concrete offers engineering attributes such as heightened production efficiency, reduced construction duration, robust resilience to external disturbances, and performance benefits including consistent product quality and precise component dimensions [10]. To expedite mold turnover and reduce curing time in factories, enhancing the early strength of precast concrete has emerged as a pressing concern [11]. Four methods are employed to enhance the early strength of concrete: (1) the utilization of special cement; (2) the utilization of emerging nano-mineral admixtures; (3) the enhancement of construction techniques and maintenance practices; (4) the incorporation of early strength admixtures [12].

Currently, the durability issues of most specialized cements remain unresolved, with their high dosages and costs further constraining their application scope. For instance, the adoption of fast-hardening cement can escalate costs by 17% to 25%, rendering it unsuitable for the widespread adoption of precast concrete. Nano-admixtures employ micro-scale control to enhance the early strength performance of UHPC. However, the current research on nano-admixtures is still in its infancy. Considering the need for stability in prefabricated products, large-scale implementation remains unrealistic. Although steam curing is a common method to ensure early strength in precast concrete, its lengthy curing time, high cost, substantial energy consumption, environmental unfriendliness, and coarse crystal size contribute to increased concrete brittleness and reduced durability [13]. Early strength agents constitute key admixtures in UHPC, markedly enhancing concrete’s early strength and expediting curing time [14]. Inorganic and organic early strength agents are commonly utilized in concrete applications. Numerous scholars have introduced various types of early strength agents into UHPC systems, yet the mechanisms of these agents within UHPC vary [15]. A singular early strength agent is insufficient to meet practical requirements. While some agents yield positive effects, they often detrimentally affect workability and operational timelines. Given the actual production conditions of prefabricated UHPC, preserving workability is paramount. Consequently, the development of suitable early strength agents to enhance cost-efficiency and performance has emerged as a crucial research avenue [16,17]. In summary, this study investigates the operational performances of two distinct early strength agents at a water/binder ratio of 0.17. It examines the influence of varied types and dosages of these agents on the mechanical properties of UHPC during room-temperature curing [18]. The isothermal calorimeter measured hydration heat release rates, while XRD/SEM analysis determined the microstructures and elemental compositions of hydration products. These analyses aimed to identify suitable components and dosages of early strength agents, optimize prefabricated UHPC construction processes, and expand UHPC’s application scope [19,20,21]. In prefabricated UHPC production, a faster setting time and accelerated concrete strength growth rate within reasonable bounds effectively reduce template removal wait times, resulting in increased output and enhanced economic benefits [22]. Hence, efficient early strength agents play a crucial role in prefabricated UHPC production [23,24].

## 2. Materials and Methods

### 2.1. Materials

The test employed various raw materials including cement, fly ash, silica fume, polycarboxylate superplasticizer, quartz sand, and steel fiber. Additionally, organic early strength agent O (Ca(HCO_2_)_2_) and inorganic early strength agent I (Al_2_(SO_4_)_3_) were used. The test utilized P.II 52.5 cement manufactured by Baiyin Zhongcai Co., Ltd. (Baiyin, China). The chemical composition of the cementitious material is detailed in Table 1. The quartz sand utilized in the experiment was sourced from Zhixiang Industrial Trading Co., Ltd. (Yongdeng, China). Table 2 presents its particle size distribution and chemical composition.

**Figure 1:**
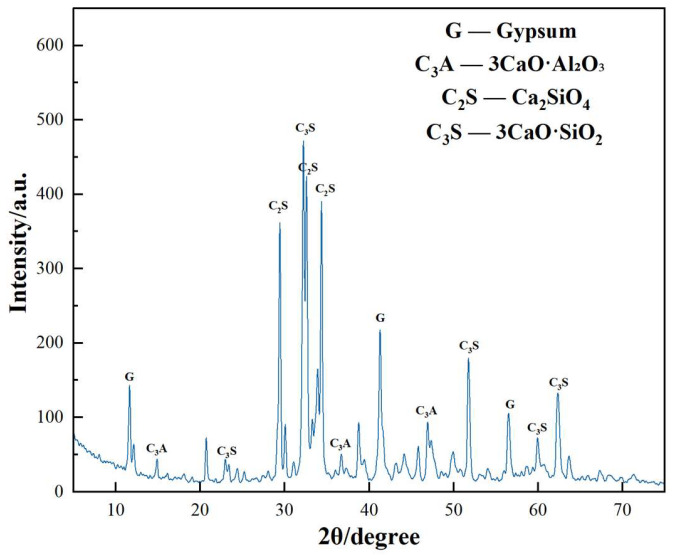
XRD pattern of cement.

**Figure 2:**
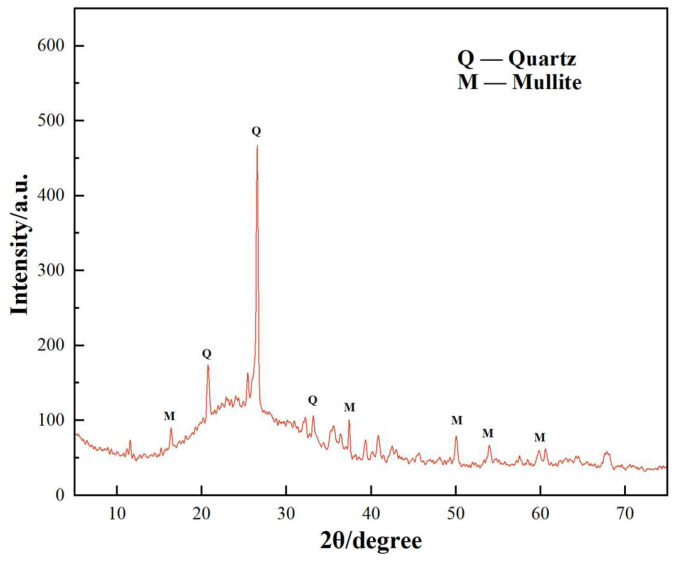
XRD pattern of fly ash.

Figure 1 illustrates the X-ray diffraction pattern of the tested cement. The figure reveals predominant phases including tricalcium silicate, dicalcium silicate, and tricalcium aluminate, aligning with the chemical analysis results. The fly ash sourced from Jingtai Mingxin Co., Ltd., (Jingtai, China) is depicted in Figure 2 through X-ray diffraction analysis. The figure highlights quartz and mullite phases, characteristic of low-calcium fly ash.

The polycarboxylate superplasticizer (SP) utilized in the test is a liquid water-reducing agent, manufactured by Jiangsu Subot New Materials Co., Ltd. (Nanjing, China), boasting a water reduction rate exceeding 35% and a solid content of 30%. Its technical specifications are detailed in Table 3. Ganzhou Daye Metal Fiber Co., Ltd. (Ganzhou, China) supplied the copper-plated micro-steel fibers for the experiment, boasting a tensile strength surpassing 2300 MPa. They measure 13 mm in length and have an equivalent diameter of 0.2 mm.

The organic early strength agent O (Ca(HCO_2_)_2_) utilized in the test was supplied by Tianjin Baishi Chemical Co., Ltd., with a purity exceeding 98%. Similarly, the inorganic early strength agent I (Al_2_(SO_4_)_3_) was provided by Fuchen (Tianjin) Chemical Reagent Co., Ltd. (Tianjin, China), with a purity exceeding 97%. Both early strength agents, in the form of white powder, were stored in laboratory dry storage boxes within their shelf lives.

All raw materials utilized in the UHPC mixture discussed in this article were chosen based on the principle of material stability while fulfilling the performance criteria.

### 2.2. Mix Design

After careful consideration of the strength and constructability of UHPC, it was ultimately determined that the water/cement ratio of UHPC would be set at 0.17, with the dosage of polycarboxylic acid superplasticizer set at 3% of the mass of the cement material. The water content of the superplasticizer was accounted for via total water consumption.

To investigate the impacts of various types and dosages of early strength agents on UHPC performance, the design mix ratios are detailed in Table 4, where the dosage of early strength agent is expressed as the mass fraction relative to the cementitious material. The designations in Table 4 are as follows: R denotes the control group without any early strength agent, O denotes the inclusion of organic early strength agent (Ca(HCO_2_)_2_), I denotes the incorporation of inorganic early strength agent (Al_2_(SO4)_3_), and OI denotes the combination of both early strength agents.

### 2.3. Specimen Molding and Maintenance

Raw materials were weighed according to the design mix ratio. Initially, the dry ingredients were introduced into the mixer and blended at a low speed of 100 rpm for 2 min to ensure uniform mixing. Subsequently, copper-plated steel fibers were slowly introduced and mixed at low speed for 4 min until evenly dispersed within the dry mixture [25]. Subsequently, stirring water and polycarboxylate water-reducing agent were concurrently added. The mixture was stirred at low speed for 3 min, followed by high-speed stirring at 200 rpm for an additional 3 min. Lastly, the early strength agent was introduced, and mixing continued at high speed for 3 min to ensure thorough contact between the raw materials, water, and water-reducing agent, facilitating the molding and curing of the slurry.

Upon the completion of mixing, the UHPC sample was poured into concrete molds measuring 100 mm × 100 mm × 100 mm and 100 mm × 100 mm × 400 mm. The former was designated for compressive testing, while the latter was allocated for bending tests. In each set, 6 blocks for compression testing and 6 blocks for bending testing were prepared. Following batch numbering, a layer of plastic film was applied to the surface of each sample to prevent excessive moisture loss and ensure sample integrity. Subsequently, the UHPC samples are placed in a curing chamber under controlled conditions, with a temperature set at 20 ± 1 °C and relative humidity maintained at 98.0%. The curing period was segmented into four intervals, 1.5 days, 3 days, 7 days, and 28 days, to facilitate subsequent performance testing.

### 2.4. Testing Methods

#### 2.4.1. Workability and Setting Time

The T/CECS 864-2021 standard was used to detect the working performances of different samples [26]. During the test, the excess ultra-high-performance concrete mixture around the slump tube is removed, and then the slump tube is lifted vertically and smoothly. When the sample no longer continues to expand or the flow time has reached 90 s, the maximum diameter of the concrete flow diameter surface and the diameter perpendicular to the maximum diameter are measured with a steel ruler.

The setting times of different samples were detected via the T/CECS 864-2021 standard. During the test, the sample cylinder is placed on the penetration resistance meter, and the end of the measuring needle is in contact with the surface of the mixture. The measuring needle should be uniformly penetrated into the mixture at a depth of 25 mm ± 2 mm within 10 ± 2 s, and the maximum penetration resistance value should be recorded, which should be accurate to 10 N. The experimental results of the setting time should be determined via the arithmetic mean value of the setting time of the three samples.

#### 2.4.2. Compressive and Flexural Strength

According to the T/CECS 864-2021 ultra-high-performance concrete test scheme standard’ and DB62/T 4690-2023 ultra-high-performance concrete application technical specification’, the mechanical properties of the UHPC specimens were tested [27]. The compressive test (100 mm × 100 mm × 100 mm cube specimens) was carried out on a microcomputer-controlled constant loading pressure testing machine with a range of 3000 kN. The bending test (prism specimens of 100 mm × 100 mm × 400 mm) was carried out on an electro-hydraulic servo universal testing machine with a maximum load of 1000 kN.

#### 2.4.3. Hydration Heat

The degree of hydration and the rate of hydration heat release were determined via the GB/T 12959 2024 cement hydration heat determination method. Relevant experiments were carried out on the cement hydration heat measurement system model PTS-12 S [28]. The PTS-12 S automatic measurement system, based on the semi-adiabatic method, was employed to quantify the heat of hydration of the UHPC. It comprises a calibration calorimeter, a super low-temperature circulating constant water bath, a high-precision temperature sensor, a data acquisition system, and eight glass thermostats (each with a volume of approximately 1.5 L). UHPC mortar samples, with constant mass ratios, were cast into these glass thermoses, which were subsequently placed in the calibrated calorimeter to measure the released heat. Throughout the test, the calorimeter was maintained in a water bath at 20 ± 0.1 °C. The temperature variations in the samples were recorded via the data acquisition system. The system’s acquisition range spans from 0 °C to 100 °C, with an accuracy of 0.1 °C. The software automatically computes the hydration heat value over a 28-day period by summing the heat accumulated in the calorimeter and the heat dissipated to the surrounding atmosphere.

In the preceding text, the total heat Q_X_ released during hydration is the combined result of the heat stored in the calorimeter and the heat dispersed to the surroundings. This value is calculated using Formula (1).

(1) $$Q_{x}=C_{P}(t_{x}-t_{0})+K\sum F_{0-x}$$

where Q_X_ represents the total heat released via UHPC hydration at a specific age (J), C_P_ represents the total heat capacity of the calorimeter after installing UHPC mortar (J/°C), t_x_ represents the temperature of the mortar at X hours of age (°C), t_0_ represents the initial temperature of the mortar at the specified age (°C), K represents the heat dissipation constant of the calorimeter (J/h·°C), and F_0−x_ represents the area between the constant temperature line of the water tank and the temperature curve from 0 to X hours (h·°C).

The heat of hydration q_x_ (J/g) of UHPC at the hydration age of X hours was computed using Formula (2), and the calculation result was rounded to 1 J/g.

(2) $$q_{x}=\frac{Q_{X}}{G}$$

where q_x_ represents the heat of hydration of UHPC at a specific age (J/g), Q_X_ represents the total heat released by the UHPC at a specific age (J), and G represents the mass of the cementitious material used in the test, measured in grams (g).

Each UHPC sample underwent a hydration heat test using two sets of calorimeters for parallel testing. If the discrepancy between the two test results was less than 10 J/g, the average value was adopted as the hydration heat result of the UHPC sample. However, if the difference exceeded 10 J/g, the test had to be repeated.

#### 2.4.4. X-ray Powder Diffraction

After the mechanical properties test was completed, the broken samples were collected and immersed in anhydrous ethanol to terminate the hydration reaction of UHPC and finally dried in an oven at 45 °C to constant weight [29]. The hydration products were tested via XRD. The scanning range was 2θ = 5°–75°, and the scanning rate was 4°/min.

#### 2.4.5. Scanning Electron Microscopy

The microstructures of raw materials and samples were analyzed via scanning electron microscopy (SEM-EDS, ZEISS, SUPRA55, Jena, Germany). Experimental conditions: beam spot range of 1.5–3.5 mm, maximum magnification of 100,000 times, vacuum degree of 8 × 10^–5^ Torr, and sample observation distance of 10–10.5 mm.

## 3. Results and Analysis

### 3.1. Impact of Early Strength Agents on Workability

**Figure 3:**
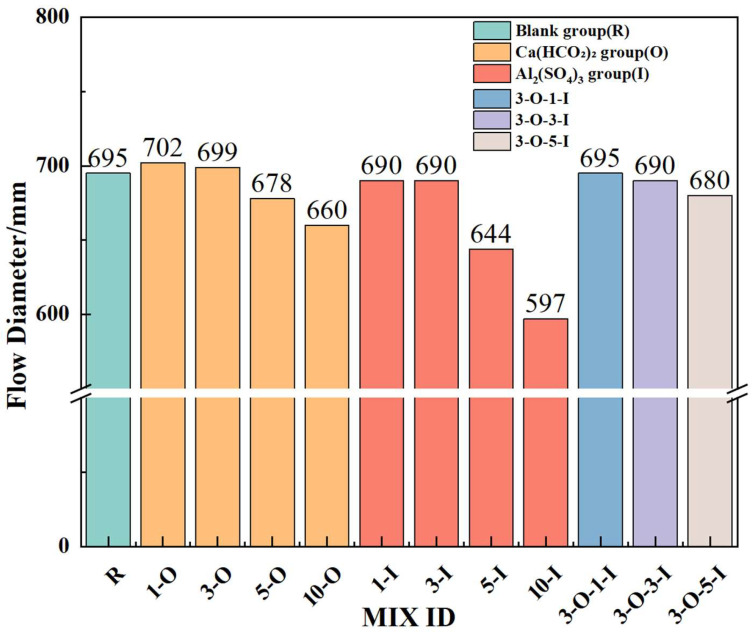
Flow diameter of UHPC with the addition of early strength agent.

This study investigated the impacts of various early strength agents on the workability of UHPC, as depicted in Figure 3. Ca(HCO_2_)_2_ and Al_2_(SO_4_)_3_ exhibit minimal impact on workability at lower dosages (≤0.3%), with a data gap of less than 1%. With increasing dosage, the workability of Ca(HCO_2_)_2_ at 0.5% and 1% concentrations decreased by 2.4% and 5.0%, respectively, compared to the control group. At Al2(SO_4_)_3_ dosages of 0.5% and 1%, workability decreased by 7.3% and 14.1%, respectively, compared to the control group. Excessive incorporation of Ca(HCO_2_)_2_ impacts workability, possibly due to a large number of formate ions chemically binding to silicon atoms, thereby increasing the system viscosity of the UHPC slurry. Excessive incorporation of Al_2_(SO_4_)_3_ adversely affects workability, possibly due to the accelerated reaction rates of C_3_A, which promotes UHPC hardening and reduces free water, resulting in decreased workability. In actual prefabricated UHPC production, early strength agent impact on UHPC performance should be controlled within 2%, with fluidity exceeding 600 mm. Inadequate fluidity may cause surface bubbles on prefabricated components, impacting production quality. Hence, caution is necessary when determining the dosage of early strength agent.

Flow diameter data for a single type of early strength agent suggest that to sustain optimal performance, Ca(HCO_2_)_2_ dosage should not exceed 0.3%, and the Al_2_(SO_4_)_3_ dosage should be limited to 0.5%. Figure 3 illustrates the impact on UHPC flow diameter when two early strength agents are included. The fluidity of 3-O-1-I remained unaffected compared to the blank group. Fluidity reductions of 0.7% and 1.4% were observed for 3-O-3-I and 3-O-5-I, respectively, all falling within acceptable limits [30,31].

### 3.2. Impact of Early Strength Agents on Setting Time

**Figure 4:**
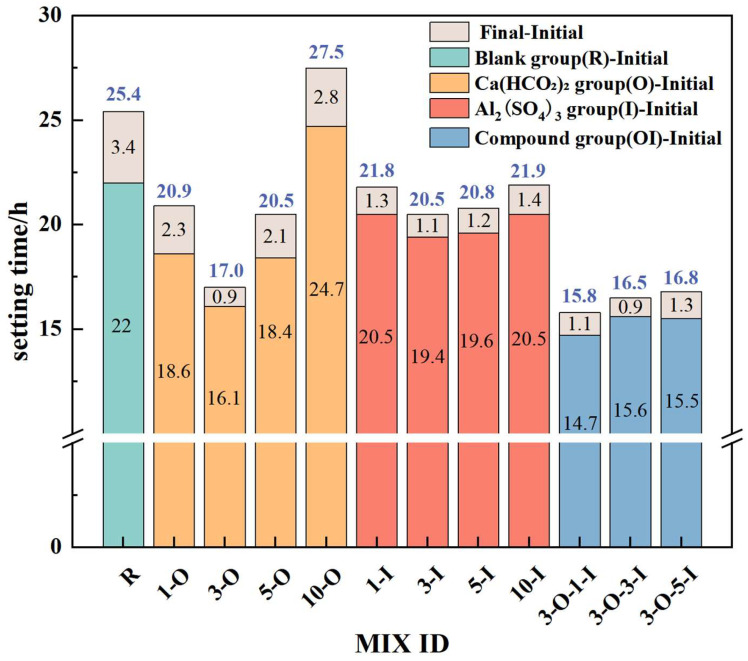
UHPC setting time data.

The setting time serves as a crucial indicator illustrating the impact of early strength. Figure 4 depicts how the utilization of two early strength agents and dosages affects the setting time of UHPC.

The blank group (R) exhibited an initial setting time of 22 h and a final setting time of 25.4 h. The initial setting times of Ca(HCO_2_)_2_ at 0.1%, 0.3%, and 0.5% were 18.6 h, 16.1 h, and 18.4 h, respectively. Compared with the control group, the initial setting times decreased by 15.4%, 26.8%, and 16.4%, respectively. The ultimate setting time also decreased, with a reduction similar in magnitude to that of the initial setting time. The initial setting time of Ca(HCO_2_)_2_ was 24.7 h at a dosage of 1%. Compared with the control group, the initial setting time increased by 12.3%. The final setting time was 27.5 h, which was 8.3% higher than that of the control group. The organic early strength agent Ca(HCO_2_)_2_ shortened the initial and final setting time of the UHPC system within a 0.5% dosage, with the best effect observed at a 0.3% dosage, while the initial and final setting time of UHPC was delayed at a 1% dosage. Calcium formate functions as an organic early strength agent. At a dosage of 1.0%, abnormal setting behavior was observed. This indicates the presence of a specific acceleration threshold for this early strength agent.

The initial setting times of Al_2_(SO_4_)_3_ at 0.1%, 0.3%, 0.5%, and 1% were 20.5 h, 19.4 h, 19.6 h, and 20.5 h, respectively. Compared with the control group, the initial setting times decreased by 6.8%, 11.8%, 10.9%, and 6.8%, respectively. The final setting times were 21.8 h, 20.5 h, 20.8 h, and 21.5 h, respectively. Compared with the control group, the setting times decreased by 14.2%, 19.3%, 18.1%, and 17.3%. The inorganic early strength agent Al_2_(SO_4_)_3_ shortens the initial and final setting times of the UHPC system at the aforementioned dosages. The impact is noticeable at dosages of 0.3% and 0.5% but diminishes at 1%. This is attributed to the excessive use of the early strength agent, which impedes gypsum dissolution and slows down the hydration rate.

The initial setting times of the composite group (OI) with 0.3% Ca(HCO_2_)_2_ and 0.1%, 0.3%, and 0.5% Al_2_(SO_4_)_3_ were 14.7 h, 15.6 h, and 15.5 h, respectively. Compared with the control group, the initial setting times decreased by 33.2%, 29.1%, and 29.5%, respectively. The final setting times were 15.8 h, 16.5 h, and 16.8 h, respectively. Compared with the control group, the setting times were reduced by 37.8%, 35.0%, and 33.9%. When Ca(HCO_2_)_2_ and Al_2_(SO_4_)_3_ are co-doped, the effect is improved compared with the control group and the single-type early strength agent. This is because HCOO^−^ can bind to silicon atoms, thereby cross-linking adjacent silicate groups, while the ionization and hydrolysis of Al_2_(SO_4_)_3_ can interact with gypsum in cement, reducing the concentration of CaSO_4_ in the slurry. It promotes the rapid reaction of C_3_A in cement, thus eliminating the stunting effect of gypsum and promoting the setting time [32]. Therefore, these two early strength agents exhibit a positive effect under reasonable dosage conditions, significantly enhancing the condensation of UHPC.

### 3.3. Impact of Early Strength Agents on Compressive and Flexural Strength

**Figure 5:**
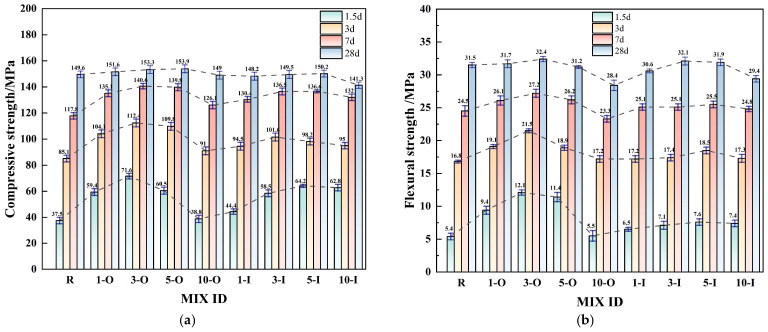
Mechanical properties of single early strength agent specimens. (**a**) Compressive Strength; (**b**) Flexural strength.

Figure 5a shows the compressive strength of the samples with different early strength agents at 1.5 days, 3 days, 7 days, and 28 days. Ca(HCO_2_)_2_ has a positive effect on the compressive strength of the sample within 0.5 percent, and the improvement effect is not obvious at 1 percent. Among them, the compressive strength at 0.5 percent dosage is lower than at 0.3 percent dosage, indicating that the optimum dosage of Ca(HCO_2_)_2_ should be within 0.5 percent. Compared to the control group (R), Al_2_(SO_4_)_3_ enhanced compressive strength significantly at 1.5 d, 3 d, and 7 d across all four dosage levels, with the most notable improvement observed at the 0.5% dosage level.

Both strength agents significantly improved the early strength of the UHPC system at the appropriate dosages. The compressive strengths at 1.5 days for samples 1-O, 3-O, 5-O, and 10-O reached 59.4 MPa, 71.6 MPa, 60.5 MPa, and 38.8 MPa, respectively, representing increases of 58.4 percent, 90.9 percent, 61.3 percent, and 3.5 percent compared to the control group (R). Under the same dosage, the compressive strengths at 1.5 days for samples 1-I, 3-I, 5-I, and 10-I reached 44.4 MPa, 58.5 MPa, 64.2 MPa, and 62.8 MPa, respectively, representing increases of 18.4 percent, 55.2 percent, 71.2 percent, and 67.5 percent compared to the control group (R). The test results indicate that the effect of Ca(HCO_2_)_2_ on improving compressive strength in the early stage was significantly better than that of Al_2_(SO_4_)_3_. This may be because Ca(HCO_2_)_2_ is weakly acidic in the system, which reduces the pH value of the system and accelerates the dissolution of gypsum, resulting in higher early hydration efficiency than Al_2_(SO_4_)_3_. In terms of medium-term strength improvement, the strength of the group mixed with Ca(HCO_2_)_2_ increased by an average of 74.6 MPa from 3 days to 7 days, representing an increase of 118.3 percent, while the strength of the group doped with Al_2_(SO_4_)_3_ increased by 78.8 MPa on average from 3 days to 7 days, representing an increase of 146.6 percent. According to the test results, Al_2_(SO_4_)_3_ has a better effect on improving compressive strength in the medium term than Ca(HCO_2_)_2_. This may be because during the hydration process of Al_2_(SO_4_)_3_, the ettringite crystal nucleus formed in the external liquid phase of the gel film can promote the rupture of the protective film and accelerate the dissociation of the clinker. At the same time, in the early stage of hydration, a large amount of Ca^2+^ is consumed to form amorphous CH, which fills the voids inside UHPC and improves the compactness of the concrete.

However, when compared with 3-O and 5-O, the strength of sample 10-O significantly decreased at 1.5 days and 3 days and slightly decreased at 7 days. This could be due to excessive Ca(HCO_2_)_2_ dissolving more gypsum at a low water/binder ratio, generating C_3_A·Ca(HCO_2_)_2_·30H_2_O, which attaches to the surface of unhydrated particles, hindering subsequent hydration and causing uneven internal hydration of concrete, thus introducing defects [33]. Compared to the control group (R), the 28-day strength of Al_2_(SO_4_)_3_ decreased at dosages of 0.1% and 1%. The smaller decrease observed at the 0.1% dosage level may be attributed to statistical error, while the more significant decrease at the 1% dosage level suggests that an excessive Al_2_(SO_4_)_3_ dosage negatively affects the later strength of UHPC. This may be due to excessive sulfate ions inducing excessive expansion of the UHPC matrix, resulting in the formation of micro-cracks that may not be readily discernible internally, thereby impacting the mechanical characteristics [34].

Figure 5b shows the flexural strengths of the specimens with different early strength agents at 1.5 days, 3 days, 7 days, and 28 days. The flexural strengths of Ca(HCO_2_)_2_ and Al_2_(SO_4_)_3_ at 0.1%, 0.3%, and 0.5% were higher than those of the control group (R), which were consistent with the compressive data. Compared with the control group (R), the flexural strength of the 10-O group decreased at 28 days, which was due to the change in pore structure caused by the uneven hydration caused by the incorporation of excessive Ca(HCO_2_)_2_, which caused fiber debonding inside the concrete to cause the flexural strength to decrease. In summary, the early strength effect of Ca(HCO_2_)_2_ is better than that of Al_2_(SO_4_)_3_ under reasonable dosage.

**Figure 6:**
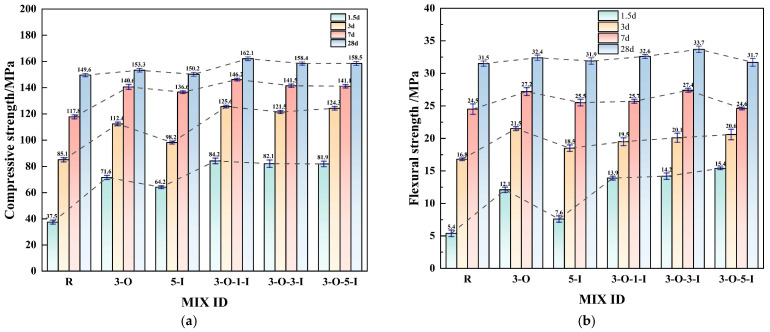
Mechanical properties of compound early strength agent specimens. (**a**) Compressive Strength; (**b**) Flexural strength.

Figure 6a displays the compressive strengths at 1.5, 3, 7, and 28 days under the influence of various early strength agents. Specifically, the 3-O and 5-I groups represent the single-doped categories demonstrating the most significant efficacy level of Ca(HCO_2_)_2_ and Al_2_(SO_4_)_3_, respectively. These groups serve as reference data for comparison purposes. The data depicted in Figure 6a indicate that the compressive strengths of the OI group surpasses those of the blank group (R), 3-O, and 5-I, suggesting a synergistic effect resulting from the combined use of the two early strength agents during hydration.

This phenomenon may stem from the introduction of aluminum sulfate (${SO}_{4}^{2-}$) into the system, facilitating the formation of AFt and secondary gypsum, while the inclusion of Ca(HCO_2_)_2_ expedites gypsum dissolution. The compressive strength of the 3-O-1-I group increased significantly compared to the blank group (R), with increments of 124.5%, 20.7%, 24.2%, and 8.4% at 1.5, 3, 7, and 28 days, respectively. Combining these two early strength agents addresses the issue of sluggish late-stage strength increase with Ca(HCO_2_)_2_ and the slow hydration rate of Al_2_(SO_4_)_3_ during the early stages, thereby optimizing the strength of the UHPC system [35].

Figure 6b depicts the flexural strength at 1.5 d, 3 d, 7 d, and 28 d under various early strength agents. Consistent with the compressive data, the OI group exhibited superior early strength compared to the blank group (R) and other singly doped groups. However, the flexural tensile strength of 3-O-5-I in the mixed group at 7 d and 28 d was notably lower than that of the 3-O-3-I. This could be attributed to an excessive amount of early strength agent, resulting in excessive hydration. The rapid formation of coarse Aft during the early hydration stage creates uneven internal hydration in concrete, leading to structural defects. Numerous AFt crystals form a dense protective layer, incorporating unhydrated cement particles, hindering subsequent hydration processes and resulting in strength reduction.

Based on the strength data, combining the aforementioned two early strength agents enhances the early strength effect. However, the overall dosage of the early strength agent should be carefully evaluated to prevent adverse effects.

### 3.4. Impacts of Early Strength Agents on Hydration Reaction Process

**Figure 7:**
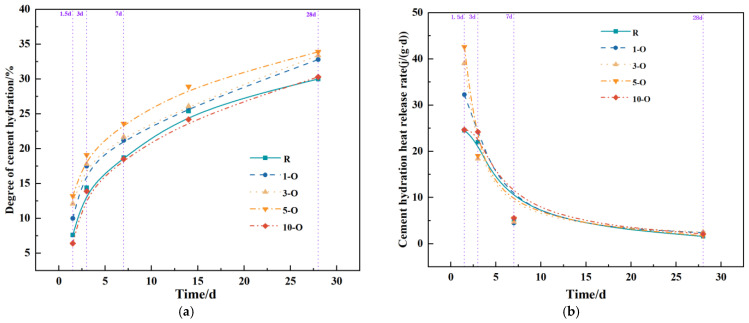
Curves of hydration with single early strength agent specimens(O). (**a**) Cement hydration degree(O); (**b**) Cement hydration heat release rate(O).

**Figure 8:**
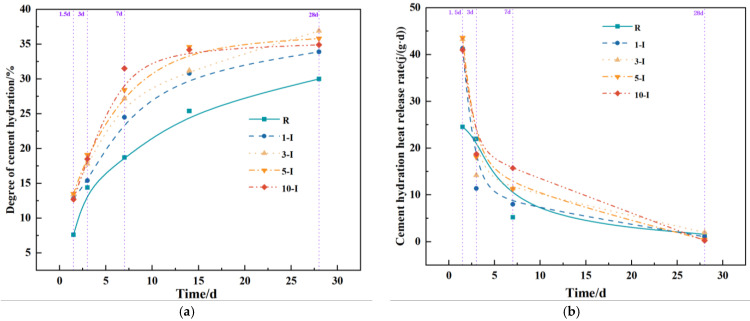
Curves of hydration with single early strength agent specimens (I). (**a**) Cement hydration degree (I); (**b**) Cement hydration heat release rate (I).

**Figure 9:**
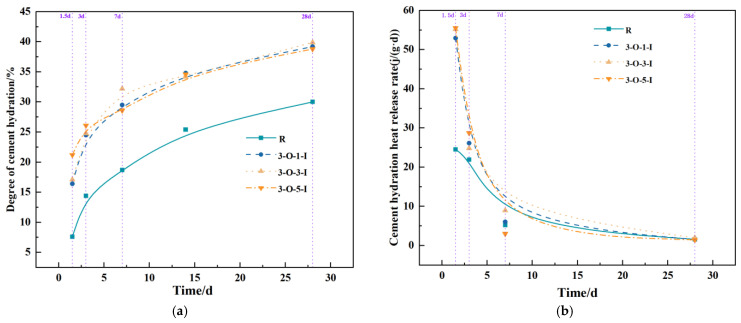
Curves of hydration with compound early strength agent specimens (OI). (**a**) Cement hydration degree (OI); (**b**) Cement hydration heat release rate (OI).

To objectively assess the impacts of various early strength agents on UHPC under different dosages, this study conducted experiments to analyze their influence on the hydration degree and rate. The hydration curves are depicted in Figure 7, Figure 8 and Figure 9. When considering Figure 7a,b, the hydration degree values of the blank group (R) at 1.5 d, 3 d, 7 d, and 28 d were 7.6%, 14.4%, 18.7%, and 30%, respectively. In comparison to the blank group (R), the hydration degree of the 1-O, 3-O, and 5-O groups improved at all stages. In particular, the effect of 5-O was pronounced, with hydration levels being 73.7%, 32.6%, 13.8%, and 13% higher than those of the blank group (R) at 1.5 d, 3 d, 7 d, and 28 d, respectively. Conversely, at a Ca(HCO_2_)_2_ content of 1%, a negative effect was observed, with the hydration degree decreasing at 1–7 d and no effect being noted at 28 d. It is evident that the inclusion of a suitable range of Ca(HCO_2_)_2_ notably increases early heat release, attributed to the finer and easier nucleation of initial precipitated AFt.

Figure 8 illustrates the impact of a single addition of the early strength agent Al_2_(SO_4_)_3_ on the hydration process of UHPC under constant temperature curing at 20 °C. In Figure 8a,b, the 1-I group, 3-I group, 5-I group, and 10-I group exhibit positive effects on the degree of hydration compared to the blank group (R). Notably, the effect of the 5-I group is the most pronounced, with the degree of hydration being 77.6%, 32.5%, 51.9%, and 19.3% higher than that of the blank group (R) at the ages of 1.5 days, 3 days, 7 days, and 28 days, respectively. The inclusion of Al_2_(SO_4_)_3_ significantly enhanced hydration during the 3–7 day period, leading to a substantial increase in heat release [36]. The data in the figure indicate that as the Al_2_(SO_4_)_3_ content increases, the hydration degree of UHPC initially rises before reaching a peak and then declines, although it does not have a detrimental impact.

The influence of Ca(HCO_2_)_2_ and Al_2_(SO_4_)_3_ on the hydration process of ultra-high-performance concrete (UHPC) is depicted in Figure 9. Analyzing Figure 9, it is evident that the hydration degree values of the 3-O-1-I group, 3-O-3-I group, 3-O-5-I group, and the blank group (R) were significantly enhanced at all ages. Notably, the effect of the 3-O-3-I group is the most pronounced, with the degree of hydration being 125.0%, 72.2%, 72.1%, and 33.0% higher than that of the blank group (R) at the ages of 1.5 days, 3 days, 7 days, and 28 days, respectively [37,38]. The enhancement in hydration degree following the combination of the two early strength agents surpasses that of both the blank group (R) and the single-mixing groups for each early strength agent (O group, I group). It is evident from the figure that compared to the blank group (R), the mixing of the two early strength agents leads to a significant increase in the early-stage heat release rate of hydration, a steady increase in hydration degree during the middle stage of hydration, and an overall increase in the final hydration degree to a certain extent.

### 3.5. Analysis of X-ray Powder Diffraction

**Figure 10:**
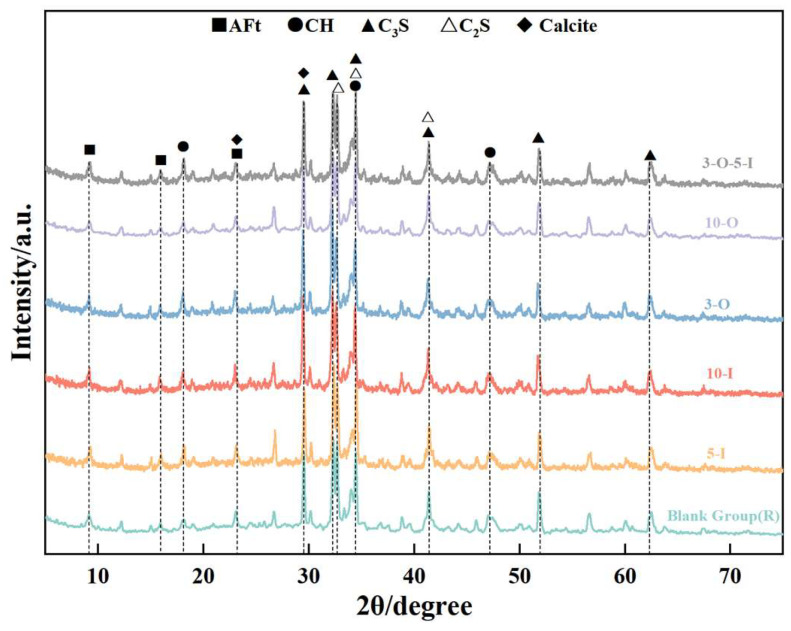
XRD patterns for 1.5 d samples.

Figure 10 illustrates the X-ray diffraction (XRD) data depicting the hydration products at 1.5 days for each group. The AFt diffraction peak gradually increases while the CH diffraction peak decreases with the increasing content of Al_2_(SO_4_)_3_ at a hydration age of 1.5 days, as depicted in the diagram. This indicates that the addition of Al_2_(SO_4_)_3_ accelerates AFt formation while also exerting a certain inhibitory effect on calcium silicate hydration. In comparison to the control group, a significant increase in the CH diffraction peak was observed upon adding 0.3% Ca(HCO_2_)_2_, suggesting the notable promotion of calcium silicate hydration at this dosage. Upon adding 1% Ca(HCO_2_)_2_, the CH diffraction peak showed no significant change compared to the control group, suggesting that the early strength effect was not pronounced at this dosage. In the compound doping group (3-O-5-I), the intensities of both AFt and CH diffraction peaks surpassed those of other groups, indicating a notable early strength effect with the combined doping of two early strength agents.

**Figure 11:**
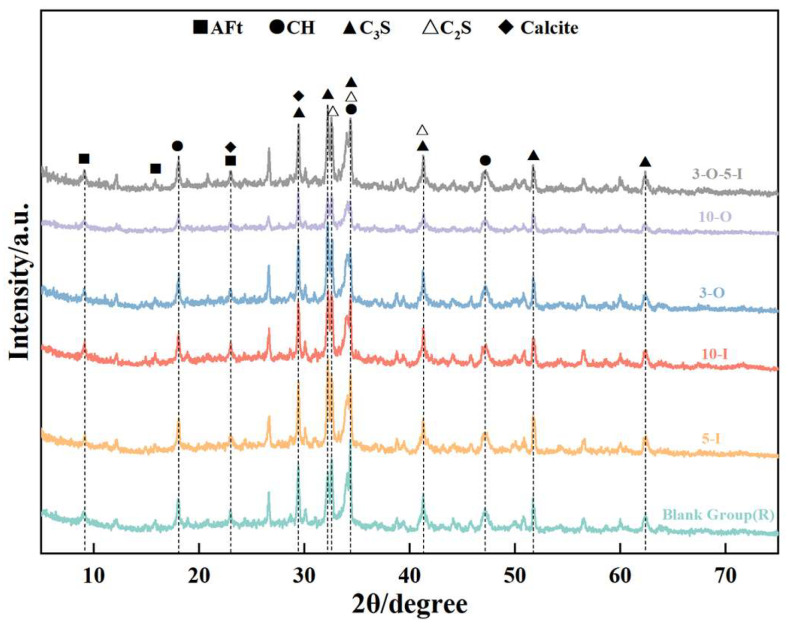
XRD patterns for 3 d samples.

Figure 11 illustrates the X-ray diffraction (XRD) data of hydration products in each group at 3 days. At a hydration age of 1.5 days, no significant difference in intensity was observed between the AFt and CH diffraction peaks of Al_2_(SO_4_)_3_ at 0.5% and 1% contents, suggesting that incremental increases in aluminum sulfate content, starting from 0.5%, do not enhance the early strength effect of UHPC. The intensities of the AFt and CH diffraction peaks with a Ca(HCO_2_)_2_ content of 1% are lower than that of the control group, suggesting that excessive Ca(HCO_2_)_2_ inhibits early strength development.

**Figure 12:**
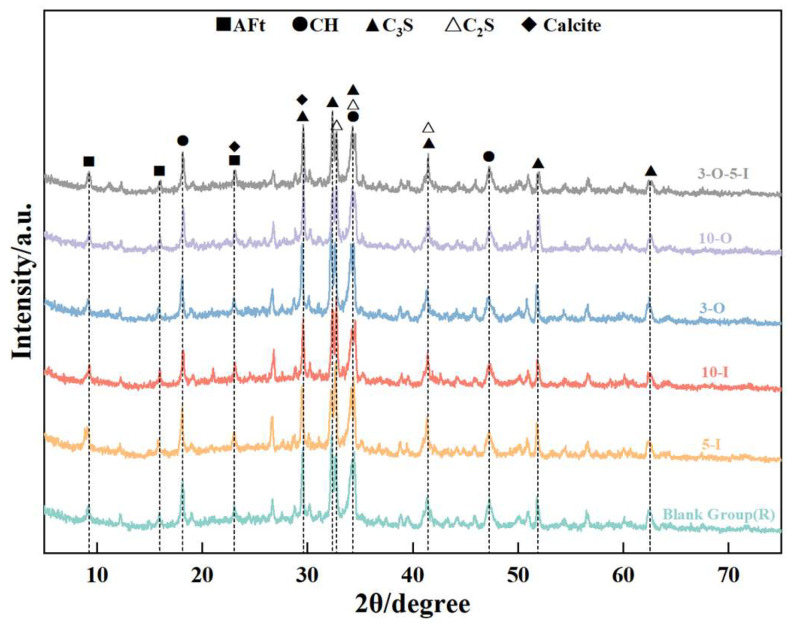
XRD patterns for 7 d samples.

Figure 12 depicts the X-ray diffraction (XRD) information of the hydration products of each group at 7 days of hydration. At this stage, the degree of hydration in each group varied. Observation of the figure reveals that with a Ca(HCO_2_)_2_ content of 1%, hydration is initially inhibited. However, by the 7-day mark, there is no significant difference in the diffraction peaks of hydration products compared to the blank group, suggesting that excessive Ca(HCO_2_)_2_ has little impact on hydration in the intermediate and late stages, consistent with the findings of strength development data. Additionally, as the amount of Al_2_(SO_4_)_3_ increases, the AFt diffraction peak remains relatively stable, while the intensity of the CH diffraction peak gradually decreases, indicating that excessive amounts lead to a reduction in the hydration rate.

**Figure 13:**
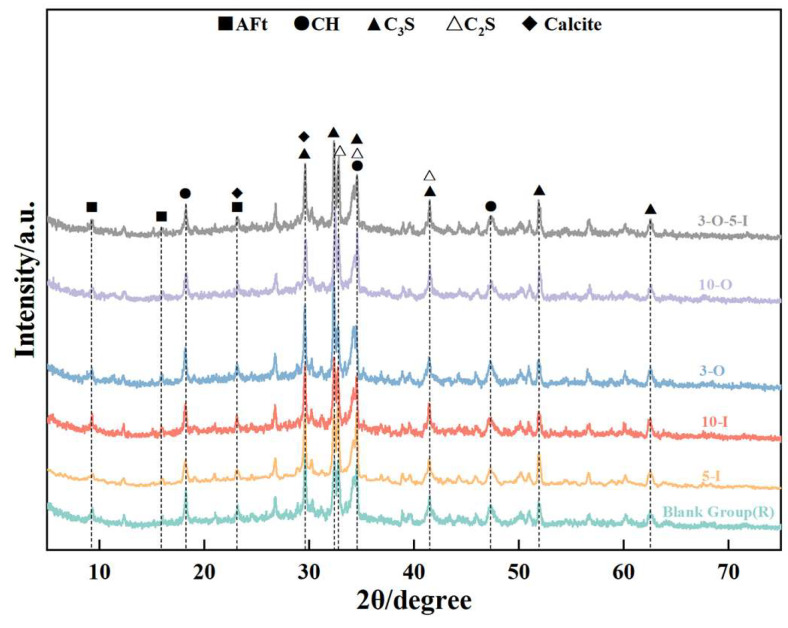
XRD patterns for 28 d samples.

Figure 13 presents the XRD information of the hydration products of each group at 28 days. By this point, hydration in each group tends to stabilize. Correlating this with Figure 12, it is evident that compared to the XRD data at 7 days, the intensity of the AFt and CH diffraction peaks progressively decreases, suggesting the transformation of some AFt to AFm. Concurrently, silica fume in the UHPC system undergoes a secondary hydration reaction (pozzolanic reaction) with the CH produced during hydration, resulting in the formation of new C-S-H gel.

### 3.6. Analysis of Scanning Electron Microscopy

**Figure 14:**
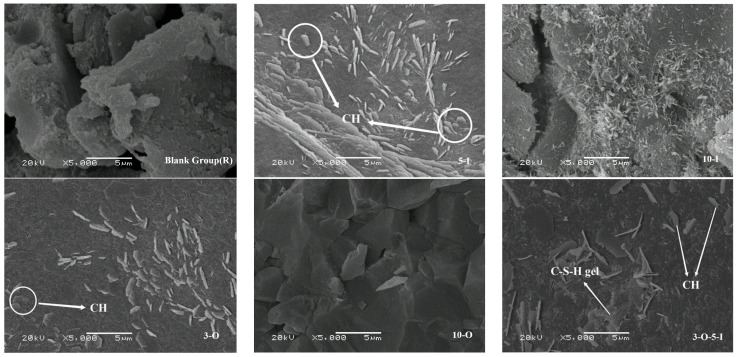
SEM images for 1.5 d samples.

The specimens aged at 1.5 days, 3 days, 14 days, and 28 days underwent observation using scanning electron microscopy (SEM). Figure 14 presents the microstructure of each group at 1.5 days of hydration. In the Al_2_(SO_4_)_3_ system, the hydration degree of ultra-high-performance concrete (UHPC) is low, as evidenced by a higher presence of unhydrated cement clinker and lesser amounts of AFt with a coarse morphology, which contributes effectively to structural stability. However, an excess of aluminum sulfate leads to observable cracks, indicating matrix expansion and detrimental effects on strength. At a Ca(HCO_2_)_2_ content of 0.3%, hexagonally shaped calcium hydroxide (CH) uniformly forms within the matrix, indicating significant promotion of calcium silicate decomposition and strength enhancement. Conversely, at a Ca(HCO_2_)_2_ content of 1%, fewer hydration products are observed compared to the other groups, with a hydration degree similar to that of the control group. Overall, a low hydration degree suggests that excessive use of early strength agents negatively impacts the hydration process. When both early strength agents are combined, AFt and CH overlap, forming a relatively stable microstructure characterized by density and the absence of obvious weak links [39].

**Figure 15:**
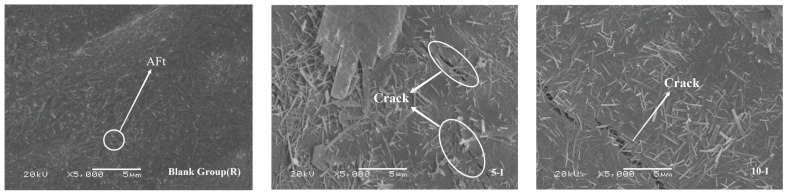

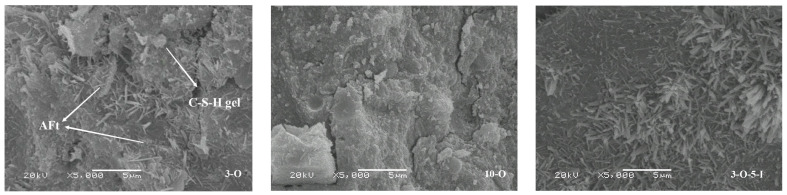
SEM images for 3 d samples.

As the duration increases, the microstructure of each sample group becomes denser. Figure 15 illustrates the microstructure of each group at 3 days. At 3 days, the hydration degree of the control group is low, with an increased presence of unhydrated clinker particles in the system. Within the images of group 10-I, it is evident that certain AFt and C-S-H compounds gather at micro-cracks, overlapping in a grid-like pattern. Throughout the hydration process, some hydration products form within the cracks, leading to varying degrees of reduction in crack width and scale. However, the cracks persist, which may significantly influence strength. At a Ca(HCO_2_)_2_ content of 1%, in comparison to the microstructure at 1.5 days, some AFt analogs begin to dissolve, allowing the hydration reaction to proceed. Nonetheless, the hydration degree remains lower than that of the control group [40].

**Figure 16:**
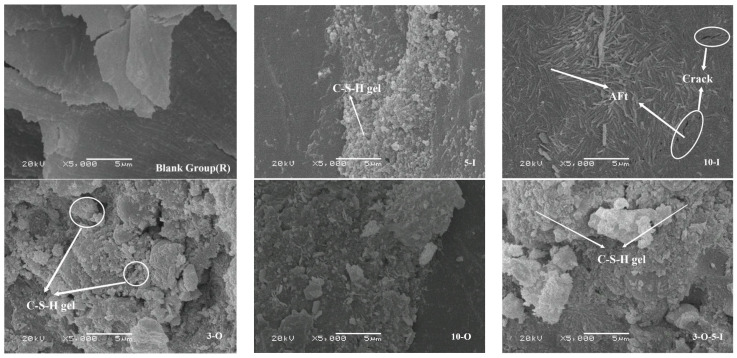
SEM images for 7 d samples.

**Figure 17:**
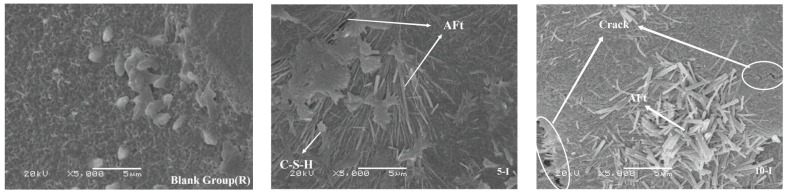

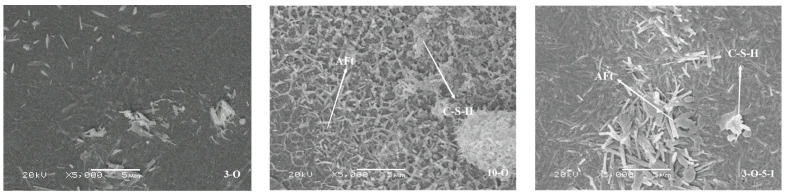
SEM images for 28 d samples.

Figure 16 and Figure 17 depict the microstructures of each group at 7 and 28 days of hydration. At 7 days, there was a notable enhancement in the compactness of the slurry within each group. The amorphous C-S-H gel and calcium hydroxide (CH) exhibited overlapping and staggered patterns, tightly encasing the unreacted clinker particles. Consequently, the spacing and gaps between the hydration products and unreacted clinker particles diminished gradually. From the diagram, it is evident that while the widths of micro-cracks in the 10-I group decreased further, the number of cracks increased.

At 28 days of age, based on the XRD data from each group, a significant decrease in the intensity of the calcium hydroxide (CH) diffraction peak was observed. Nevertheless, microscopic examination of each group revealed the persistence of CH hexagonal crystals, which developed into laminated plates and were seamlessly integrated with the calcium–silicate–hydrate (C-S-H) gel, as well as unreacted clinker and fly ash particles. This reduction in the total amount of CH diminished the weak links within the slurry, thereby enhancing the durability and stability of the structure.

In addition to the 10-I group, it was observed that in each group, the vitreous body and a portion of the calcium hydroxide (CH) on the surface of fly ash particles underwent consumption due to the pozzolanic reaction [40]. Consequently, a newly generated grid-like flocculent gel tightly adhered to the particle surfaces, establishing a strong connection with the surrounding hydration products.

There is no obvious weak interface in the image [41]. The overall number of hydration substances in the 10-I group is large, but due to the existence of micro-cracks, the microstructure has serious defects, especially the fact that the number of hydration products that overlap across the cracks is small, which further reduces its matrix strength. It is worth noting that there is no significant difference in the microstructure and hydration degree of hydration products between the 10-O group and the other groups at the age of 28 d, indicating that the excessive Ca(HCO_2_)_2_ mainly affects the early stage of hydration and has little effect on the late stage of hydration.

## 4. Conclusions

This study focuses on the influence of different dosages of two kinds of early strength agent on the early performance of UHPC under the condition of a 0.17 water/binder ratio. According to the results of this study, the following conclusions can be drawn:

(1) Within a reasonable dosage range, both early strength agents, Ca(HCO_2_)_2_ and Al_2_(SO_4_)_3_, can substantially reduce the curing time and enhance the mechanical properties of UHPC. The optimal dosages of Ca(HCO_2_)_2_ and Al_2_(SO_4_)_3_ are approximately 0.3% and 0.5%, respectively.
(2) With the exception of 10-O, all other groups significantly enhanced hydration during the early and middle stages. The heat release rate of Al_2_(SO_4_)_3_ during these stages exceeds that of Ca(HCO_2_)_2_.
(3) The two early strength agents did not change the type of hydration products but changed the shape and quantity of the hydration products, especially in the early stage of hydration, which greatly increased the content of AFt. In addition, the two early strength agents improved the microstructure and matrix density at reasonable dosages.
(4) Adding calcium formate or calcium sulfate alone does not meet the expected early strength effect. The sole addition of calcium formate exhibits the drawback of sluggish mid-term hydration, whereas the exclusive use of aluminum sulfate fails to adequately enhance initial strength. Moreover, using a large dosage of a single early strength agent poses risks to UHPC. However, when these two agents are combined, they exhibit a synergistic effect, resulting in better performance than that obtained using either agent alone.

## Figures and Tables

**Table 1:** Main chemical composition of raw materials.

Materials	SiO_2_	Al_2_O_3_	Fe_2_O_3_	CaO	MgO	K_2_O	Na_2_O	SO_2_	CO_2_	Total
Cement/%	13.58	5.37	3.51	57.05	1.12	0.82	0.62	16.25	0.00	98.32
Fly ash/%	56.30	27.99	4.99	6.38	0.75	1.18	0.82	0.00	0.22	98.63
Silica fume/%	96.25	0.33	0.54	0.52	0.31	1.87	0.18	0.00	0.00	100.00

**Table 2:** Quartz sand particle size range and chemical composition analysis.

Particle Size Range/mm	SiO_2_ Content/%	Fe_2_O_3_ Content/%	Moisture Content/%
0.15–1.00	99.0–99.6	0.03–0.10	1.5

**Table 3:** Technical indicators of polycarboxylate superplasticizer.

Water Reduction/%	Methane Content/%	Solid Content/%	Fluidity of Cement Mortar/mm
≥35	≤6.0	30%	≥240

**Table 4:** UHPC mix ratio (kg/m^3^).

Numbering	Cement	Flyash	Silica Fume	Quartz Sand	Steel Fiber	Water	Accelerating Admixture/%	
							O	I
R	780	220	150	950	188	171	/	/
1-O	780	220	150	950	188	171	0.1	/
3-O	780	220	150	950	188	171	0.3	/
5-O	780	220	150	950	188	171	0.5	/
10-O	780	220	150	950	188	171	1.0	/
1-I	780	220	150	950	188	171	/	0.1
3-I	780	220	150	950	188	171	/	0.3
5-I	780	220	150	950	188	171	/	0.5
10-I	780	220	150	950	188	171	/	1.0
3-O-1-I	780	220	150	950	188	171	0.3	0.1
3-O-3-I	780	220	150	950	188	171	0.3	0.3
3-O-5-I	780	220	150	950	188	171	0.3	0.5

## Data Availability

The data presented in this study are available upon request from the corresponding author (L.H.).
